# Supplementary material for: Risk Factors for Influenza-Induced Exacerbations and Mortality in Non-Cystic Fibrosis Bronchiectasis
Source: Viruses. 2023 Feb 14;15(2):537. doi: 10.3390/v15020537 (PMC9961441; doi:10.3390/v15020537)
Supplement: Supplementary file 1 [file viruses-15-00537-s001.zip › viruses-2200531-supplementary.pdf]

Supplementary Materials

Table S1. Baseline characteristics of bronchiectasis patients with influenza infection (PCR or rapid antigen test).

	All	Clinic§	Hospitalization	
	n = 231	n = 73	n = 158	p-Value
Age	67.5 ± 14.5	60.7 ± 13.9	70.7 ± 13.7	<.001
Sex (Female)	113 (48.9%)	42 (57.5%)	71 (44.9%)	0.075
Etiology				
Post infection (TB)	27 (11.7%)	9 (12.3%)	18 (11.4%)	0.837
Post infection (other)	141 (61.0%)	35 (48.0%)	106 (67.1%)	0.006
Immunodeficiencies	6 (2.6%)	1 (1.4%)	5 (3.2%)	0.668
Idiopathic	23 (10.0%)	15 (20.6%)	8 (5.1%)	<.001
Previous exacerbation*	1.1 ± 1.6	0.4 ± 1.2	1.4 ± 1.7	<.001
0	107 (46.3%)	57 (78.1%)	50 (31.7%)	<.001
1	66 (28.6%)	11 (15.1%)	55 (34.8%)	
2	36 (15.6%)	2 (2.7%)	34 (21.5%)	
≥ 3	22 (9.5%)	3 (4.1%)	19 (12.0%)	
BACI index	8.3 ± 6.2	6.8 ± 5.9	8.9 ± 6.2	0.015
Comorbidity				
Solid malignancy	15 (6.5%)	2 (2.7%)	13 (8.2%)	0.154
COPD	125 (54.1%)	33 (45.2%)	92 (58.2%)	0.065
Liver disease	59 (25.5%)	23 (31.5%)	36 (22.8%)	0.158
Connective tissue disease	13 (5.6%)	2 (2.7%)	11 (7.0%)	0.237
Diabetes	68 (29.4%)	20 (27.4%)	48 (30.4%)	0.644
Chronic renal disease	44 (19.0%)	12 (16.4%)	32 (20.3%)	0.492
Cardiovascular disease	71 (30.7%)	20 (27.4%)	51 (32.3%)	0.455
Pulmonary function				0.636
FEV_1 < 50	32 (13.9%)	9 (12.3%)	23 (14.6%)	
FEV_1: 50_80	28 (12.1%)	11 (15.1%)	17 (10.8%)	
FEV_1 > 80	48 (20.8%)	20 (27.4%)	28 (17.7%)	
FVC: < 80 pred. %	38 (16.5%)	13 (17.8%)	25 (15.8%)	
Influenza test				0.557
PCR	14 (6.1%)	3 (4.1%)	11 (7.0%)	
Rapid antigen test	217 (93.9%)	70 (95.9%)	147 (93.0%)	
Influenza medicine				0.362
Amantadine	1 (0.4%)	1 (1.4%)	0 (0.0%)	
Zanamivir	39 (16.9%)	11 (15.1%)	28 (17.7%)	
Oseltamivir	185 (80.1%)	58 (79.5%)	127 (80.4%)	
Peramivir	6 (2.6%)	3 (4.1%)	3 (1.9%)	
Medicine after 1 month				
Systemic Steroid	96 (41.6%)	10 (13.7%)	86 (54.4%)	<.001

Antibiotic	160 (69.3%)	7 (9.6%)	153 (96.8%)	<.001

Note: BACI, bronchiectasis aetiology comorbidity index; COPD, chronic obstruction pulmonary disease; FEV_1, forced expiratory volume in one second; FVC, forced vital capacity; pred.; predicted value; TB, tuberculosis.[§] The clinic group was defined as those with no episode of hospitalization. * Previous exacerbation within one year

Table S2. Clinical characteristics of bronchiectasis and influenza-related (PCR or rapid antigen test) hospitalization.

	All Ward<br>n = 158	Non-Critical<br>n = 129	Respiratory Failure<br>n = 29	p-Value
Age	70.7 ± 13.7	71.8 ± 12.7	65.7 ± 17.1	0.082
Sex (Female)	71 (44.9%)	58 (45.0%)	13 (44.8%)	0.990
Etiology				
Post infection (TB)	18 (11.4%)	14 (10.9%)	4 (13.8%)	0.746
Post infection (other)	106 (67.1%)	91 (70.5%)	15 (51.7%)	0.051
Immunodeficiencies	5 (3.2%)	4 (3.1%)	1 (3.5%)	1.000
Idiopathic	8 (5.1%)	6 (4.7%)	2 (6.9%)	0.640
Previous exacerbation*	1.4 ± 1.7	1.3 ± 1.5	1.7 ± 2.4	0.464
0	50 (31.7%)	45 (34.9%)	5 (17.2%)	0.027
1	55 (34.8%)	38 (29.5%)	17 (58.6%)	
2	34 (21.5%)	30 (23.3%)	4 (13.8%)	
≥ 3	19 (12.0%)	16 (12.4%)	3 (10.3%)	
BACI index	8.9 ± 6.2	8.9 ± 6.3	9.1 ± 6.0	0.895
Comorbidity				
Solid malignancy	13 (8.2%)	10 (7.8%)	3 (10.3%)	0.708
COPD	92 (58.2%)	78 (60.5%)	14 (48.3%)	0.229
Liver disease	36 (22.8%)	27 (20.9%)	9 (31.0%)	0.241
Connective tissue disease	11 (7.0%)	9 (7.0%)	2 (6.9%)	1.000
Diabetes	48 (30.4%)	36 (27.9%)	12 (41.4%)	0.154
Chronic renal disease	32 (20.3%)	23 (17.8%)	9 (31.0%)	0.110
Cardiovascular disease	51 (32.3%)	42 (32.6%)	9 (31.0%)	0.874
Pulmonary function				0.643
FEV_1 < 50	23 (14.6%)	18 (14.0%)	5 (17.2%)	
FEV_1: 50_80	17 (10.8%)	13 (10.1%)	4 (13.8%)	
FEV_1 > 80	28 (17.7%)	25 (19.4%)	3 (10.3%)	
FVC: < 80 pred. %	25 (15.8%)	21 (16.3%)	4 (13.8%)	
Influenza test				0.119
PCR	11 (7.0%)	7 (5.4%)	4 (13.8%)	
Rapid antigen test	147 (93.0%)	122 (94.6%)	25 (86.2%)	
Influenza medicine				0.607
Amantadine	0 (0.0%)	0 (0.0%)	0 (0.0%)	
Zanamivir	28 (17.7%)	24 (18.6%)	4 (13.8%)	
Oseltamivir	127 (80.4%)	103(79.8%)	24 (82.8%)	
Peramivir	3 (1.9%)	2 (1.6%)	1 (3.4%)	

Note: BACI, bronchiectasis aetiology comorbidity index; COPD, chronic obstruction pulmonary disease; FEV_1, forced expiratory volume in one second; FVC, forced vital capacity; pred., predicted value; PCR, polymerase chain reaction; TB, tuberculosis. * Previous exacerbation within one year.

Table S3. Clinical parameters and main clinical outcomes of influenza-related (PCR or rapid antigen test) hospitalization.

	All Ward n = 158	Non-Critical n = 129	Respiratory Failure n = 29	p-Value
Sputum culture in ward	110 (69.6%) 85 (65.9%)		25 (86.2%)	0.032
Negative	75 (68.2%)	63 (74.1%)	12 (48.0%)	0.014
Pseudomonas aeruginosa	13 (11.8%)	10 (11.8%)	3 (12.0%)	1.000
Klebsiella pneumoniae	5 (4.6%)	4 (4.7%)	1 (4.0%)	1.000
Haemophilus influenzae	5 (4.6%)	3 (3.5%)	2 (8.0%)	0.318
Fungus	8 (7.3%)	3 (3.5%)	5 (20.0%)	0.015*
NTM	2 (1.8%)	1 (1.2%)	1 (4.0%)	0.405
Staphylococcus aureus	2 (1.8%)	0 (0.0%)	2 (8.0%)	0.050
MDR-AB	4 (3.6%)	2 (2.4%)	2 (8.0%)	0.222
Lab Data				
WBC ($\times 10^3$ /ul)	9.7 ± 4.3	9.4 ± 3.9	11.2 ± 5.7	0.111
Platelet ($\times 10^3$ /ul)	191.9 ± 69.6	193.2 ± 65.7	186.2 ± 86.2	0.683
C-reactive protein (mg/L)	81.7 ± 95.5	62.8 ± 67.9	139.9 ± 138	0.008
Creatinine，baseline (mg/dL)	1.4 ± 1.7	1.3 ± 1.8	1.6 ± 1.7	0.360
Creatinine，ward (mg/dL)	1.5 ± 2.0	1.3 ± 1.8	2.2 ± 2.3	0.031
Acute kidney injury	35 (22.2%)	23 (17.8%)	12 (41.4%)	0.006
Influenza medicine				0.607
Amantadine	0 (0.0%)	0 (0.0%)	0 (0.0%)	
Zanamivir	28 (17.7%)	24 (18.6%)	4 (13.8%)	
Oseltamivir	127 (80.4%)	103 (79.8%)	24 (82.8%)	
Peramivir	3 (1.9%)	2 (1.6%)	1 (3.5%)	
Other medication				
Systemic Steroid	86 (54.4%)	65 (50.4%)	21 (72.4%)	0.031
Antibiotic	153 (96.8%)	125 (96.9%)	28 (96.6%)	1.000
Inhalation gentamicin	4 (2.5%)	1 (0.8%)	3 (10.3%)	0.02*
Days of hospitalization	12.5 ± 12.5	9.7 ± 9.8	25.1 ± 15.4	<.001
Shock	19 (12.0%)	5 (3.9%)	14 (48.3%)	<.001*
In-hospital mortality	12 (7.6%)	6 (4.7%)	6 (20.7%)	0.010*
30-day mortality	10 (6.3%)	5 (3.9%)	5 (17.2%)	0.019*
3-month mortality	13 (8.2%)	6 (4.7%)	7 (24.1%)	0.003*
One-year outcomes				
Mortality	11 (7.53%)	7 (5.69%)	4 (17.39%)	0.073
Respiratory failure	12 (7.53%)	8 (5.69%)	5 (17.39%)	0.012*
Severe exacerbations	13 (7.53%)	9 (5.69%)	6 (17.39%)	0.009
Mild and severe exacerbations	1.84 ± 2.83	1.71 ± 2.81	2.57 ± 2.87	0.183

Note: MDR-AB, multidrug-resistant Acinetobacter baumannii; NTM, non-tuberculosis mycobacteria; WBC, white blood cell count.

Table S4. The Harrell's C-index of the significant parameters in Table 4 and Table 5.

**Table 4 Parameters**	**Harrell's C-Index**	**Table 5 Parameters**	**Harrell's C-Index**
Age	0.61	Age	0.62
Female	0.53	Female	0.57
Etiology		Etiology	
Post infection	0.58	Tuberculosis	0.54
Idiopathic	0.56	Cardiovascular disease	0.54
Previous exacerbations*	0.7	Platelet count	0.58
0		Acute kidney injury	0.68
1		Systemic steroid	0.52
2		Respiratory failure	0.57
≥ 3		Shock	0.75
BACI index	0.62		
Pulmonary function	0.58		
FEV_1: < 50 pred.%			
FEV_1: 50-80 pred. %			
FEV_1: > 80 pred. %			
FVC: <80 pred. %			

Note: BACI, bronchiectasis aetiology comorbidity index; FEV_1, forced expiratory volume in one second, FVC, forced vital capacity. * Previous exacerbation within one year.
